# Radiomics approach based on biphasic CT images well differentiate “early stage” of adrenal metastases from lipid-poor adenomas: A STARD compliant article

**DOI:** 10.1097/MD.0000000000030856

**Published:** 2022-09-23

**Authors:** Lixiu Cao, Wengui Xu

**Affiliations:** a Department of Molecular Imaging and Nuclear Medicine, Tianjin Medical University Cancer Institute and Hospital, National Clinical Research Center for Cancer, Tianjin Key Laboratory of Cancer Prevention and Therapy, Tianjin’s Clinical Research Center for China, Tianjin, China; b Department of ECT, Tangshan People’s Hospital, Tangshan, China.

**Keywords:** abdominal contrast enhanced computed tomography, “early stage” adrenal metastases, lipid-poor adenomas, radiomics

## Abstract

The aim of the study was to develop an optimal radiomics model based on abdominal contrast-enhanced computed tomography (CECT) for pre-operative differentiation of “early stage” adrenal metastases from lipid-poor adenomas (LPAs). This retrospective study included 188 patients who underwent abdominal CECT (training cohort: LPAs, 68; metastases, 64; validation cohort: LPAs, 29; metastases, 27). Abdominal CECT included plain, arterial, portal, and venous imaging. Clinical and CECT radiological features were assessed and significant features were selected. Radiomic features of the adrenal lesions were extracted from four-phase CECT images. Significant radiomics features were selected using the least absolute shrinkage and selection operator (LASSO) and multivariable logistic regression. The clinical-radiological, unenhanced radiomics, arterial radiomics, portal radiomics, venous radiomics, combined radiomics, and clinical-radiological-radiomics models were established using a support vector machine (SVM). The DeLong test was used to compare the areas under the receiver operating characteristic curves (AUCs) of all models. The AUCs of the unenhanced (0.913), arterial (0.845), portal (0.803), and venous (0.905) radiomics models were all higher than those of the clinical-radiological model (0.788) in the testing dataset. The AUC of the combined radiomics model (incorporating plain and venous radiomics features) was further improved to 0.953, which was significantly higher than portal radiomics model (*P* = .033) and clinical-radiological model (*P* = .009), with the highest accuracy (89.13%) and a relatively stable sensitivity (91.67%) and specificity (86.36%). As the optimal model, the combined radiomics model based on biphasic CT images is effective enough to differentiate “early stage” adrenal metastases from LPAs by reducing the radiation dose.

## 1. Introduction

Adrenal incidentalomas (AIs) are commonly discovered for other clinical reasons, and their frequency is increasing owing to the wider application of radiological examinations.^[1,2]^ Among AIs, adrenal adenomas are the most common benign tumors, and about 30% of adrenal adenomas are lipid-poor (>10 Hounsfield units [HU] on pre-enhanced CT value [CT-pre]).^[3–7]^ Meanwhile, the adrenal gland is also a frequent site of metastatic disease. With a history of cancer and small (<4 cm) hyperattenuating (CT-pre > 10HU) homogenous unilateral AIs, it is still quite difficult to diagnose correctly by current routine imaging examination because of atypical and overlapping radiological features.^[8–11]^ Therefore, it is a major clinical challenge to reliably differentiate early stage adrenal metastases from lipid-poor adenomas (LPAs) in small unilateral AIs.

CT washout is a reliable imaging standard for differentiating adenomas and metastases, with relatively high sensitivity and specificity.^[12–15]^ However, the disadvantages of performing adrenal washout CT should be considered, including a long delay scan, additional radiation hazards, medical cost, and lack of sensitivity.^[16–21]^ On the other hand, chemical-shift MRI can improve the diagnosis rate of LPA^[8,9,22]^; but approximately 10–20% of LPAs remain indeterminate.^[8,10]^ Moreover, not every patient has high-quality chemical-shift imaging.^[23–31]^ Both functional adenomas and metastases can show high 18-fluorodeoxyglucose uptake^[32]^ on PET/CT. Furthermore, PET/CT is not generally used before contrast-enhanced computed tomography (CECT) in most institutions, and the waiting time takes several days to undergo PET/CT. Finally, some patients may undergo unnecessary surgical resection or biopsy^[33,34]^ to achieve diagnostic certainty.

Early adrenal metastases often lack typical conventional radiological features. Thus, there is a need for the development of a noninvasive and easy method based on initial CECT to effectively distinguish early stage adrenal metastases from LPAs to guide further treatments. Hence, radiomics features have emerged as a new tool to help detect and diagnose both common and rare diseases, as well as monitor therapy, especially in oncology.^[35–37]^

With regard to adrenal lesions, CT texture analysis has been found to be effective in distinguishing benign from malignant lesions. However, validation of these findings is hampered by small samples, unspecified types of adrenal lesions, or non-detailed comparative analysis of different models.^[7,38–40]^ However, to the best of our knowledge, the specific effectiveness of radiomics models based on abdominal CECT in identifying early stage (small, unilateral, hyperattenuating, and homogenous) adrenal metastases is not known. Therefore, this study aimed to develop an optimal radiomics model based on initial CECT for differentiating LPAs from metastases in small unilateral AIs.

## 2. Materials and Methods

### 2.1. Patients

**Figure. 1:**
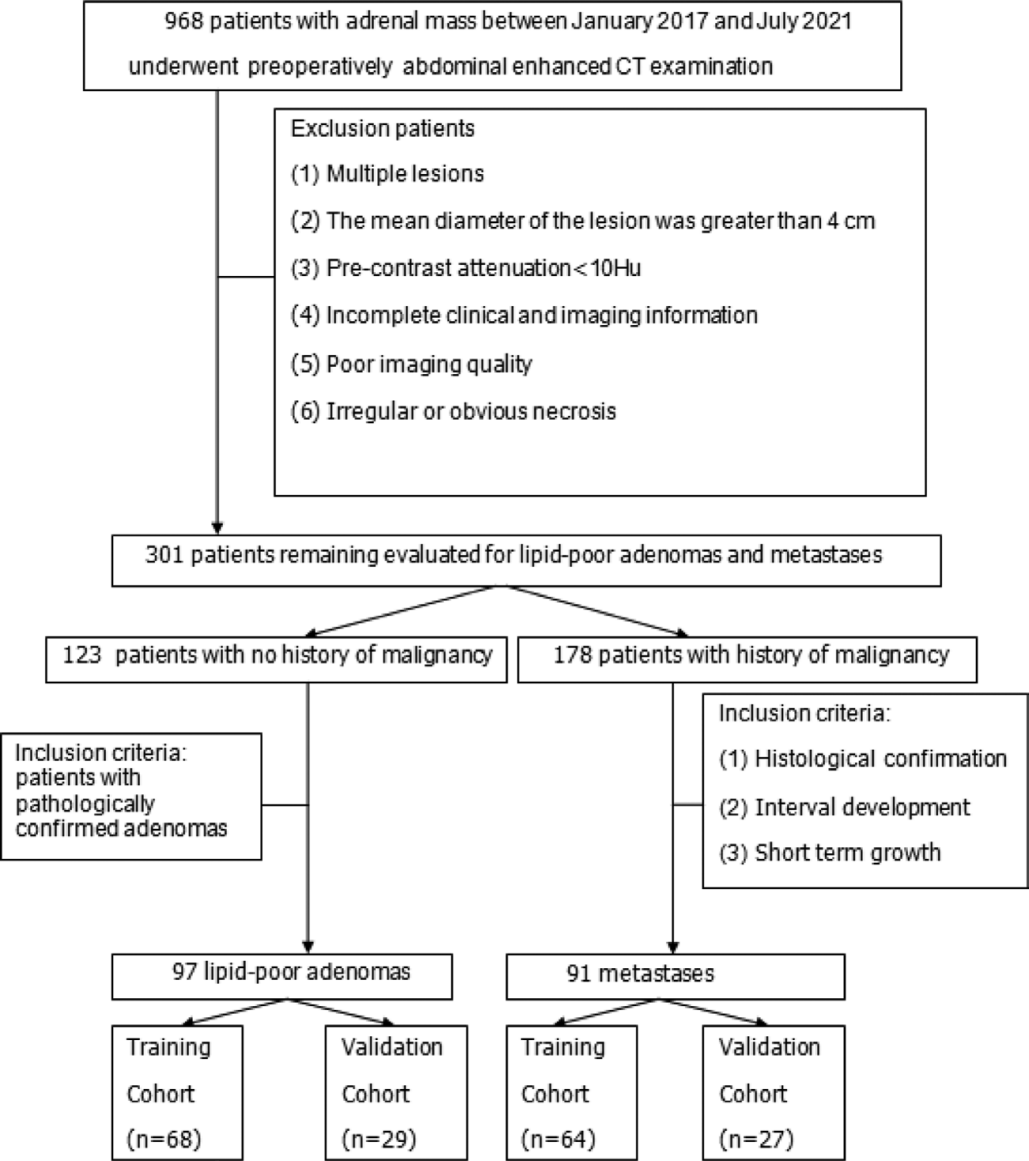
Flowchart shows the patient selection process, along with the inclusion and exclusion criteria.

This study was approved by the institutional ethics review board. Patients who underwent abdominal CECT examination with the diagnostic terms “adrenal nodule or mass” or “adrenal adenoma” or “adrenal metastasis” from January 2017 to July 2021 at our hospital were included. The following inclusion criteria were used: a small unilateral lesion (<4 cm) and a CT-pre > 10HU; complete clinical and imaging information; and regular and homogenous. If the patient had a history of cancer pathology before undergoing CECT, the following three eligibility criteria for the diagnosis of adrenal metastasis were used: needle biopsy or resection of specimen histologically confirmed (n = 7) and; when compared with previous CT scan that showed a normal adrenal gland, interval development of an adrenal nodule (n = 50); and short-term interval (<6 mo) growth^[41]^ of the nodule in the same patient (n = 34). After the pre-operative abdominal CECT, all LPAs were surgically excised and histopathologically assessed. Our study included 188 patients with AIs, comprising 97 LPAs and 91 metastases. Patients were randomly assigned to the training cohort (n = 132; 68 LPAs, 64 metastases) and validation cohorts (n = 56; 29 LPAs, 27 metastases) at a ratio of 7:3 (Fig. 1).

Age, sex, and primary malignant tumors of metastasis were recorded (Table 1).

**Table 1:** Clinical and radiological characteristics of the patients in the training and validation cohorts.

Characteristics	Training cohort (n = 132)				Validation cohort (n = 56)				P* value
	Total (n = 132)	LPAs (n = 68)	Metastasis (n = 64)	*P* value	Total (n = 56)	LPAs (n = 29)	Metastasis (n = 27)	*P* value	
Gender				.841				.977	.443
Male	67 (50.76)	36 (52.94)	35 (54.69)		25 (44.64)	13 (44.83)	12 (44.44)		
Female	65 (49.24)	32 (47.06)	29 (45.31)		31 (55.36)	16 (55.17)	15 (55.56)		
Age (yr)	60.91 ± 9.14	59.66 ± 9.85	62.05 ± 8.28	.183	60.93 ± 8.39	60.73 ± 10.49	61.13 ± 5.83	.879	.990
LD (cm)	1.91 ± 0.75	1.90 ± 0.75	1.92 ± 0.74	.893	1.83 ± 0.62	1.78 ± 0.63	1.88 ± 0.60	.601	.539
SD (cm)	1.54 ± 0.61	1.57 ± 0.61	1.52 ± 0.61	.725	1.48 ± 0.51	1.40 ± 0.52	1.48 ± 0.50	.986	.541
Lesion location				.305				.945	.232
Right	62 (46.97)	29 (42.65)	33 (51.56)		21 (37.50)	11 (37.93)	10 (37.04)		
Left	70 (53.03)	39 (57.35)	31 (48.44)		35 (62.50)	18 (62.07)	17 (62.96)		
CT-pre (HU)	33.69 ± 10.20	29.82 ± 9.70	37.2 ± 9.34	<.001	34.37 ± 10.16	28.36 ± 10.05	39.88 ± 6.47	<.001	.707
CT-a (HU)	65.39 ± 17.13	68.90 ± 19.80	62.20 ± 13.51	.051	64.85 ± 18.36	67.82 ± 21.28	62.13 ± 14.68	.304	.862
CT-p (HU)	77.61 ± 16.91	80.20 ± 16.51	72.25 ± 16.92	.137	75.98 ± 16.39	78.68 ± 17.77	73.50 ± 14.58	.295	.585
CT-v (HU)	66.57 ± 15.68	61.62 ± 11.00	71.07 ± 17.79	.001	65.22 ± 14.09	59.63 ± 13.45	70.33 ± 12.65	.009	.610
Primary tumor pathology									
Lung			39				16		
Hepatocellular carcinoma			5				2		
Gastric cancer			7				1		
Colon			6				2		
Pancreatic cancer			3				2		
Esophageal cancer			2				1		
Appendiceal cancer			1				0		
Testicular carcinoma			0				1		
Breast			1				2		

Data are numbers of patients, with percentages in parentheses.

CT-a = arterial-phase CT value, CT-p = portal-phase CT value, CT-pre = pre-enhanced CT value, CT-v = venous-phase CT value, HU = Hounsfield units, LD = long diameter, LPAs = lipid-poor adenomas, SD = short diameter.

*P* value < .05 indicates a significant difference between LPAs and metastasis in the training or validation cohort.

P* value < .05 indicates a significant difference between the training and validation cohorts.

### 2.2. Abdominal CECT protocol

The patients underwent plain and phasic CECT scans from the diaphragmatic dome to the inferior margin of the liver on GE Discovery CT750 HD spiral CT. Using a high-pressure injector, 80 to 100 mL nonionic contrast medium iopamidol (350 mg I/mL) was injected intravenously at a rate of 3 to 4 mL/second. The arterial-phase scan was automatically triggered after 6 second when the CT value of the abdominal aorta reached or exceeded 120 HU, and then the portal-phase and venous-phase scans were performed at intervals of 26 second and 120 second, respectively. Image reconstruction with section thickness and spacing of 1.25 mm was performed.

### 2.3. Radiological features

The size, four-phase CT values, and location (right or left) of the lesions were measured and assessed by two radiologists with five and nine years of experience in abdominal CECT. Long diameter (LD) and short diameter (SD) was measured in the maximum cross-section of the AIs. The region of interest encompassing two-thirds of the nodule’s maximum axial area was placed to avoid the inclusion of adjacent fat. All results were approved by consensus.

### 2.4. Radiomics feature extraction and selection

**Figure. 2:**
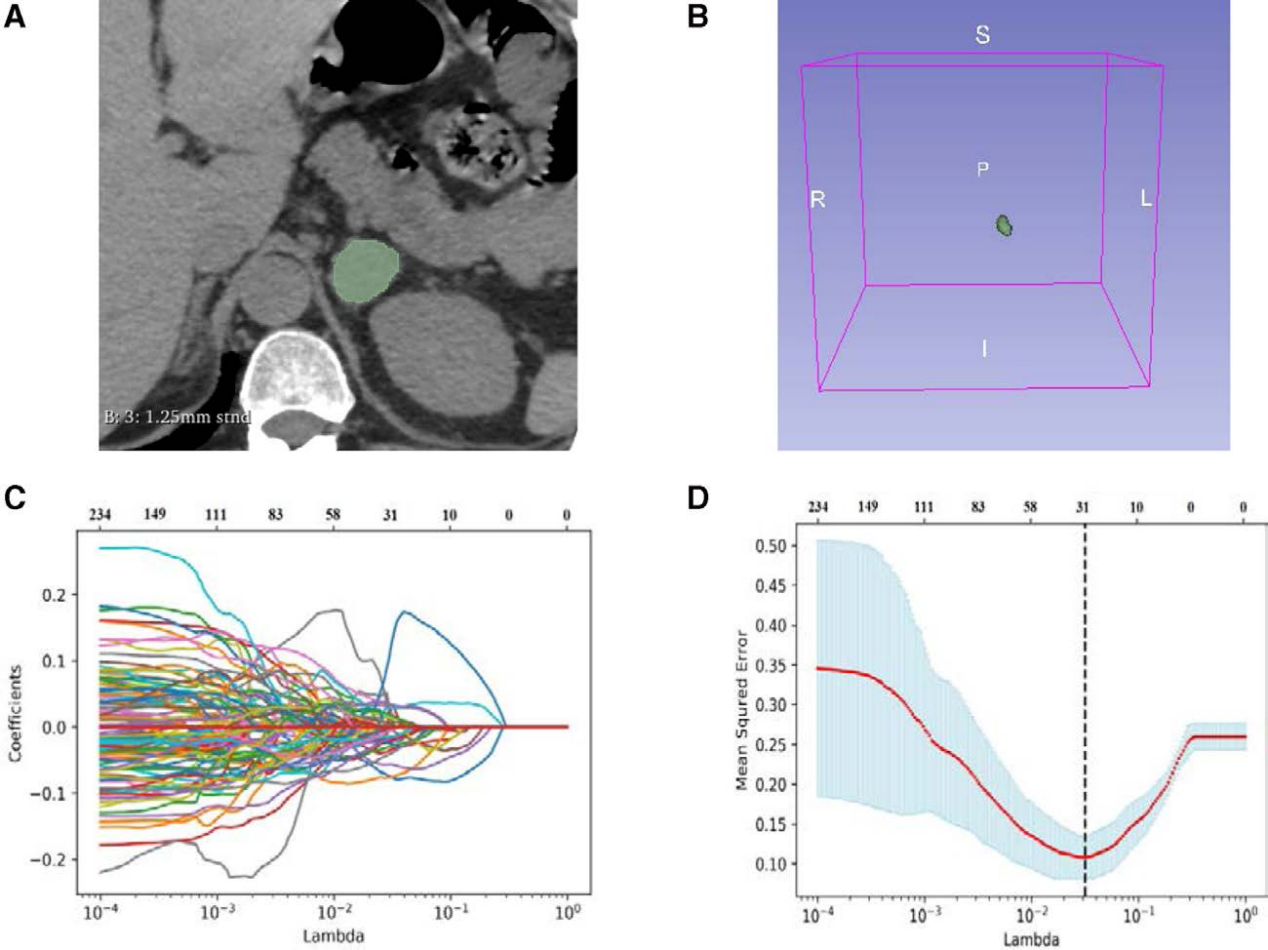
Delineation of VOI and selection of radiomic features. (a) Delineation of intratumoral region in the unenhanced CT images. (b) Three dimensional VOI of adrenal mass. (c) LASSO coefficient profiles (y-axis) of the combined radiomics features. The lower x-axis indicated the log lambda (λ). The top x-axis has the average numbers of predictors. (d) 30 combined radiomics features were selected into the LASSO model by adjusting lambda to minimize the mean square error. LASSO = least absolute shrinkage and selection operator, VOI = volume of interest.

The volume of interests (VOIs) of AIs were delineated manually by two radiologists with four and seven years of experience in each phase image (unenhanced, arterial, portal, and venous) using 3D Slicer 4.11.20210226 software (https://www.slicer.org). The entire lesion were encompassed, while extratumoral structures were carefully avoided (Fig. 2a). The reproducibility of the delineation of the VOIs by the two radiologists was assessed. The VOIs (Fig. 2b) delineated by a radiologist with seven years of experience were selected for subsequent radiomics analysis.

Radiomics features were processed and extracted by SlicerRadiomics, which is an extension using SuperBuild to build a separate library, pyradiomics, and a dependent scripted module. Using Laplacian of Gaussian filters (sigma = 1.0, 2.0, 3.0, 4.0, 5.0) and the wavelet, radiomics features can be calculated on the original or pre-processed images. When the resampling voxel dimensions were 1 × 1 × 1 mm^3^ and the intensity bin width was 25, the feature was calculated. Radiomics features of the VOIs of the AIs in each phase were extracted, such as texture features, shape, and first-order statistics. Texture features were composed of a gray-level size zone matrix, neighboring gray tone difference matrix, gray level co-occurrence matrix, gray level dependence matrix, and gray level run-length matrix. To preliminarily screen for significant radiomics features, an independent samples *t* test was employed, which reduced the dimensionality and redundancy of the features by least absolute shrinkage and selection operator (LASSO) in the training dataset (Fig. 2c). The value of the penalization parameter lambda (λ) was selected by 5-fold cross validation. The optimal features were selected by adjusting the lambda to minimize the mean square error of the model (Fig. 2d). The useful features of the VOIs of the AIs with non-zero coefficients were extracted in each phase.

### 2.5. Model construction and validation

The collinearity of clinical characteristics and radiological features selected by the independent-samples *t* test or the chi-square test and radiomics features selected by LASSO were tested based on the training dataset. Highly collinear features were deleted, and independent predictors were selected using multivariate logistic regression. Seven models were established using independent predictors to differentiate LPAs from metastasis using a support vector machine (SVM): clinical-radiological, unenhanced radiomics, arterial radiomics, portal radiomics, venous radiomics, combined radiomics, and clinical-radiological-radiomics models. A clinical-radiological-radiomics model was developed by incorporating clinical-radiological features and combined radiomics features. In both the training and validation datasets, the area under the receiver operating characteristic curve (AUC) was used to evaluate the diagnostic ability of the models and the DeLong test was used to compare the AUCs of all models.

### 2.6. Statistical analysis

Statistical analyses were performed using IBM SPSS Statistics 21.0 (https://www.ibm.com/analytics/spss-statistics-software), Python (version 3.8, https://www.python.org/), and MedCalc (version 20.026, https://www.medcalc.org/). Categorical variables and quantitative parameters were compared using the chi-square test and the independent-samples *t* test or Mann–Whitney *U* test, respectively. The intraclass correlation coefficient (ICC) was used to evaluate the inter-observer reproducibility of the feature extraction. ICC <0.5 indicated low, 0.5 to 0.79 medium, and ≥0.8 high consistency. The “sklearn.linear_model” package was used to implement LASSO regression in Python software. The correlation between variables was calculated using the “pandas” package, and correlation coefficient >0.7 were deleted. Multivariate logistic regression was implemented using backward stepwise variable selection. SVM was performed using the “sklearn” package in Python software. The correlation coefficient matrix of selected features was presented as a heatmap using the packages of “seaborn” and “matplotlib” in Python software. Differences in the AUCs between the various models were evaluated using the Delong test in MedCalc. Statistical significance was set at *P* < .05.

## 3. Results

### 3.1. Clinical and radiological characteristics of the patients

Between the training and testing datasets, no significant differences were found in any of the variables (sex, age, LD, SD, location, CT-pre, arterial-phase CT value, portal-phase CT value [CT-p], and venous-phase CT value [CT-v]) (Table 1, *P* > .05), indicating that it was reasonable to group the total data randomly. CT-pre and CT-v showed significant differences in the training and testing datasets between LPAs and metastatic patients (Table 1, *P* < .05).

### 3.2. Clinical-radiological model construction

The clinical-radiological model was constructed using an SVM by integrating CT-pre (HU) and CT-v (HU), which were independent predictors for differentiating LPAs from metastasis based on the training dataset. The AUCs of clinical-radiological model were 0.797 [95% CI: 0.707–0.869] for training dataset and 0.788 [95% CI: 0.642–0.894] for testing dataset, respectively (Table 2).

**Table 2:** Performance of different models for differentiating LPAs from metastasis.

	Training cohort (n = 132)				Validation cohort (n = 56)			
Machine learning (SVM)	AUC (95% CI)	Sensitivity	Specificity	Accuracy	AUC (95% CI)	Sensitivity	Specificity	Accuracy
Clinical-radiological model	0.797 (0707–0.869)	89.09%	62.00%	70.48%	0.788 (0.642–0.894)	87.50%	59.09%	69.57%
Unenhanced model	0.982 (0.934–0.998)	98.18%	92.00%	93.33%	0.913 (0.792–0.976)	87.50%	86.36%	82.61%
Arterial model	0.985 (0.939–0.999)	90.91%	98.00%	94.29%	0.845 (0.708–0.934)	83.53%	86.36%	84.78%
Portal model	0.965 (0.910–0.991)	98.18%	82.00%	86.67%	0.803 (0.659–0.905)	95.83%	68.18%	76.09%
Venous model	0.971 (0.918–0.994)	94.55%	94.00%	93.33%	0.905 (0.782–0.972)	83.33%	90.91%	86.96%
Combined radiomics model	0.992 (0.950–1.000)	92.70%	100.00%	96.19%	0.953 (0.846–0.993)	91.67%	86.36%	89.13%

AUC = area under the receiver operating characteristic curve, CI = confidence interval, SVM = support vector machine.

### 3.3. Radiomics feature extraction and selection

A total of 1316 quantitative features were extracted from the unenhanced, arterial, portal, and venous phases. The interobserver ICCs of the radiomics features were <0.5, 0.5 to 0.79, and ≥0.8 for 1%, 8%, and 91%, respectively, which indicated that feature extraction reproducibility was satisfactory. Fifteen features in unenhanced phase, 21 features in arterial phase, 18 features in portal phase, 23 features in venous phase and 30 combined features (15 unenhanced features and 15 venous features) (Fig. 2d) in the training dataset, were selected by LASSO, respectively. The unenhanced (n = 6), arterial (n = 8), portal (n = 7), venous (n = 6) and combined (6 unenhanced and 3 venous features) radiomics models were established by SVM after deleting highly collinear features and sequentially reducing the dimensionality using multivariate logistic regression, respectively (Table 3). The selected radiomics features had low correlation coefficients, as shown in heatmaps (Fig. 3a–e); thus, they were suitable for developing models for differentiating LPAs from metastasis.

**Table 3:** Selected radiomics features in the unenhanced, arterial, portal, venous and combined radiomics models.

The unenhanced model	The arterial model	The portal model	The venous model	The combined radiomics model
Original_shape_ Elongation	Original_shape_ Flatness	Original_shape_ Flatness	Original_shape_ Flatness	*Original_shape_ Elongation
log-sigma-1-0-mm-3D_gldm_ DependenceEntropy	log-sigma-1-0-mm-3D_firstorder_Range	log-sigma-3-0-mm-3D_glszm_ LowGrayLevelZoneEmphasis	log-sigma-5-0-mm-3D_firstorder_ Entrop	*log-sigma-1-0-mm-3D_gldm_ DependenceEntropy
Wavelet-LLH_glcm_Idn	log-sigma-5-0-mm-3D_firstorder_ InterquartileRange	log-sigma-4-0-mm-3D_gldm_SmallDependenceHighGrayLevelEmphasis	Wavelet-LLH_ gldm_ DependenceEntropy	*Wavelet-LLH_ glcm_Idn
Wavelet-LLH_gldm_ DependenceEntropy	Wavelet-LLH_ firstorder_90Percentile	log-sigma-4-0-mm-3D_glszm_ SmallAreaHighGrayLevelEmphasis	Wavelet-HHL_ glcm_ InverseVariance	*Wavelet-LLH_ gldm_ DependenceEntropy
Wavelet-LHH_glszm_ SmallAreaLowGrayLevelEmphasis	Wavelet-LLH_glcm_Imc1	log-sigma-5-0-mm-3D_glrlm_ShortRunEmphasis	Wavelet-LLL_ firstorder_ 10Percentile	*Wavelet-LHH_ glszm_ SmallAreaLowGrayLevelEmphasis
Wavelet-LLL_ firstorder_Median	Wavelet-LLH_ glszm_SizeZoneNonUniformityNormalized	Wavelet-LLH_gldm_DependenceEntropy		*Wavelet-LLL_ firstorder_Median
	Wavelet-LLH_glszm_ SmallAreaLowGrayLevelEmphasis	Wavelet-LLH_ glszm_ GrayLevelNonUniformityNormalized		#Original_shape_ Flatness
	Wavelet-LLL_glcm_ MaximumProbability			#original_firstorder_10Percentile
				#log-sigma-5-0-mm-3D_Gldm_ DependenceEnt

* The unenhanced radiomics;

# The venous radiomics

**Figure. 3:**
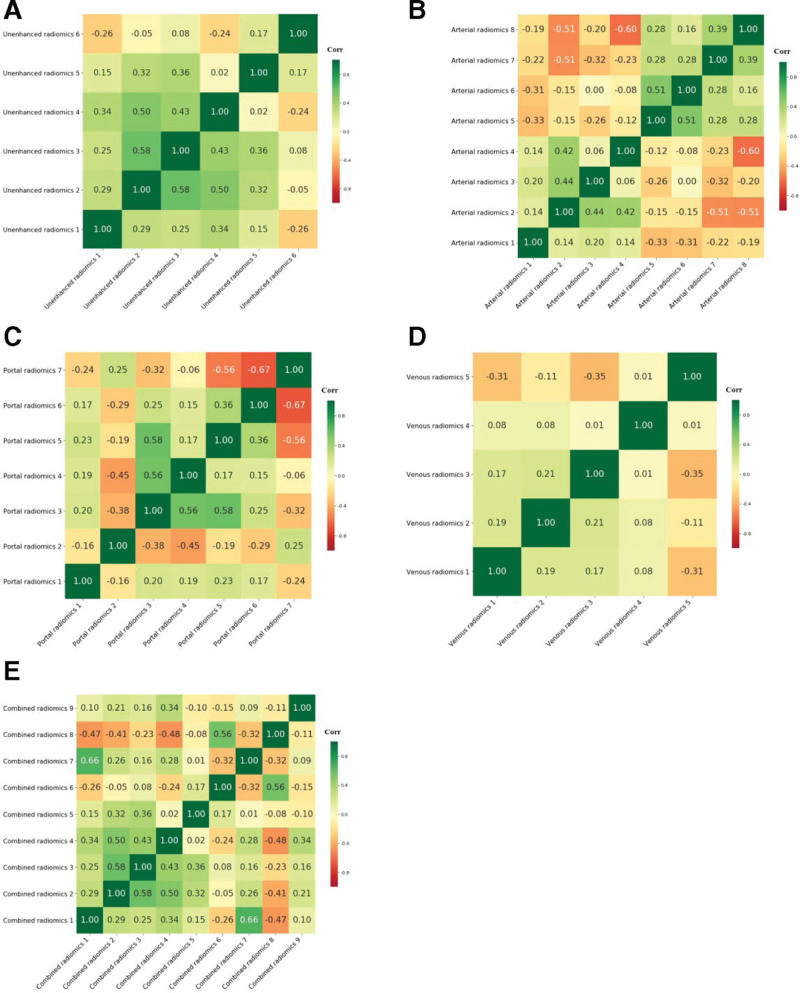
Radiomics heatmaps. (a) Heatmap depicting correlation coefficients matrix of 6 selected features in the unenhanced radiomics model. (b) Heatmap depicting correlation coefficients matrix of 8 selected features in the arterial radiomics model. (c) Heatmap depicting correlation coefficients matrix of 7 selected features in the portal radiomics model. (d) Heatmap depicting correlation coefficients matrix of 5 selected features in the venous radiomics model. € Heatmap depicting correlation coefficients matrix of 9 selected features in the combined radiomics model.

### 3.4. Development and validation of radiomics and clinical-radiological-radiomics models

During multivariate logistic regression, the clinical-radiological-radiomics model only incorporated combined radiomics features, while all clinical-radiological characteristics were excluded. Table 2 shows the performance of all models in differentiating LPAs from metastasis using SVM. In the training dataset, the combined radiomics model showed the highest AUC of 0.992, followed by the arterial (0.985), unenhanced (0.982), venous (0.971), portal models (0.965), respectively, while the AUC of the clinical-radiological model was only 0.797. However, in the testing dataset, the combined radiomics model still had the highest AUC (0.953), followed by the unenhanced (0.913), venous (0.905), arterial (0.845), portal (0.803), and clinical-radiological models (0.788). The combined radiomics model also showed the highest accuracy and a relatively high sensitivity and specificity in both training (accuracy, 96.19%; sensitivity, 92.70%; specificity, 100%) and testing datasets (accuracy: 89.13%, sensitivity: 91.67%, specificity: 86.36%) (Table 2).

### 3.5. Comparison between various models

**Figure. 4:**
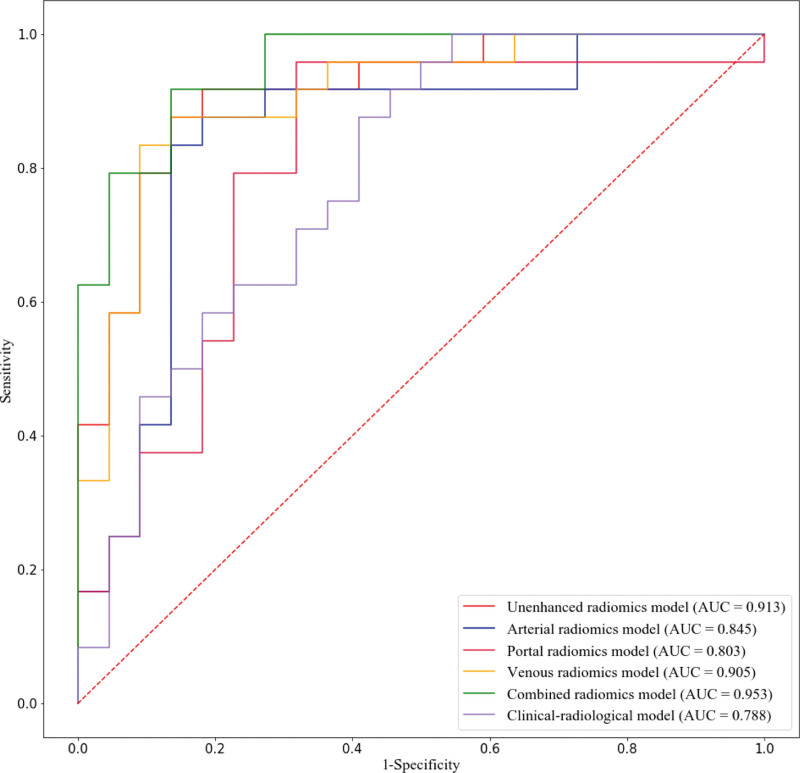
ROCs of the clinical-radiological, unenhanced, arterial, portal, venous and combined radiomics models in the validation cohort using support vector machine (SVM).

The AUC of the combined radiomics model was significantly higher than that of the clinical-radiological (*P* = .009) and portal radiomics model (*P* = .033). However, no significant differences were observed in the AUCs between other comparisons (*P* > .05) (Table 4, Fig. 4).

**Table 4:** Comparison of performance of the clinical and radiomics models in the validation cohort.

Models	AUC	Z statistic	*p*
Combined radiomics model vs Clinical-radiological model	0.953 vs 0.788	2.622	.009
Combined radiomics model vs Unenhanced model	0.953 vs 0.913	1.152	.249
Combined radiomics model vs Arterial model	0.953 vs 0.845	1.693	.090
Combined radiomics model vs Portal model	0.953 vs 0.803	2.127	.033
Combined radiomics model vs Venous model	0.953 vs 0.905	1.457	.145
Unenhanced model vs Clinical-radiological model	0.913 vs 0.788	1.596	.111
Unenhanced model vs Arterial model	0.913 vs 0.845	1.015	.310
Unenhanced model vs Portal model	0.913 vs 0.803	1.694	.090
Unenhanced model vs Venous model	0.913 vs 0.905	0.161	.872
Arterial model vs Clinical-radiological model	0.845 vs 0.788	0.608	.543
Arterial model vs Portal model	0.845 vs 0.803	0.762	.446
Arterial model vs Venous model	0.845 vs 0.905	0.917	.359
Portal model vs Clinical-radiological model	0.803 vs 0.788	0.145	.885
Portal model vs Venous model	0.803 vs 0.905	1.502	.133
Venous model vs Clinical-radiological model	0.905 vs 0.788	1.543	.123

AUC = area under the receiver operating characteristic curve.

## 4. Discussion

Our study developed and compared several radiomics and clinical-radiological models based on conventional abdominal CECT for preoperative identification of early stage adrenal metastases (small hyperattenuating homogenous unilateral) from LPAs. This study concluded that the clinical-radiological model had a lower discrimination ability than all radiomics models. The unenhanced radiomics model had the best discrimination value among all the single-phase radiomics models. Compared with all single-phase radiomics models, the performance of the combined radiomics model had additional value. Therefore, the use of unenhanced and venous radiomics features is effective enough to differentiate early stage adrenal metastases from LPAs, meanwhile, the radiation dose of biphasic CT can be reduced by omitting the arterial and portal scan.

When using CT, tumor heterogeneity and size have been found to be consistently useful features between malignant and benign adrenal masses. For instance, previous reports from Ho et al,^[39]^ Tu et al,^[40,42]^ and Schieda et al^[43]^ found that malignant adrenal tumors were larger and more heterogeneous than benign ones. In our study, large masses (mean diameter of the lesion >4 cm) and heterogeneous lesions (obvious necrosis) were excluded; therefore, LD and SD showed no significant differences between L-PAA and ASSM. In addition, we reached the same conclusion as that of Ho et al,^[39]^ who revealed that unenhanced CT attenuation was statistically significant between malignant and benign adrenal masses. As for CECT attenuation, recent studies had different views, possibly because of the difference in the enhanced scanning phases and the different proportions of primary lesions of adrenal metastases.^[40,44]^ One study by Tu et al^[40]^ reported that the attenuation of metastases and adenomas at about 70 second after injecting contrast agent (approximately similar to the portal phase of our study) did not differ significantly. Meanwhile, CT-v (HU) (approximately 3 min) rarely reported was statistically significant variable in our study, and then we used CT-pre (HU) and CT-v (HU) as independent predictors for preoperative differentiating early stage adrenal metastases from LPAs in clinical-radiological model achieving an AUC of 0.788 in testing dataset. However, the clinical-radiological-radiomics model excluded CT-pre (HU) and CT-v (HU), indicating that the differential diagnostic value of clinical-radiological features was significantly lower than that of radiomic features.

All radiomics models showed good differential diagnostic value in differentiating early stage adrenal metastases from LPAs, especially the combined radiomics model. A recent study by Ho et al^[39]^ in 2019 showed that the CECT texture features yielded a mean AUC of 0.8 and could be used as a potential method to distinguish between malignant and benign lesions; however, the study included a small sample of 20 patients and only compared 21 s-order texture features on unenhanced CT and single-phase CECT. Moreover, the malignant lesions were not all metastases but with two cases of adrenal cortical carcinomas. Another study by Andersen et al^[38]^ in 2021 indicated that for patients with history of lung cancer, some texture parameters could statistically significantly distinguish metastatic from benign adrenal masses, although they developed a combined model, but with a relatively low AUC (0.73), specificity (77%), sensitivity (58%) and accuracy (68%). In the Moawad et al study,^[7]^ the developed radiomic model showed an AUC of 0.85 with higher sensitivity (84.2%) than Andersen’s; however, the small sample of 40 cases was the main defect of the study. Furthermore, there were many types of adrenal lesions in the enrolled patients, such as adenomas, oncocytoma, and adrenal metastases. To the best of our knowledge, no previously published study has determined whether the radiomics approach can differentiate early stage adrenal metastases from LPAs based on conventional abdominal CECT and explored the optimal model by comparing different radiomic models and clinical-radiological models. The results of our study revealed that the combined radiomics model was superior to all the other models for differentiating early stage adrenal metastases from LPAs, and was significantly higher than the portal radiomics model and clinical-radiological model.

The combined radiomics model included six unenhanced and three venous radiomics features in the current study, which indicates that biphasic CT can accurately differentiate early stage adrenal metastases from LPAs and reduce the radiation dose by omitting the arterial and portal scans. Moreover, if biphasic images can reliably identify early stage adrenal metastases on initial CECT, the waiting time, medical costs, and radiation exposure for achieving diagnostic certainty will be avoided because additional imaging examinations are no longer required. In addition, compared with the 15-min delay scan of adrenal CT, biphasic CT is more likely to be widely used in clinical practice because of the reduced scanning time. Therefore, we hypothesized that the 15-min delay scan of adrenal CT could be replaced by the venous phase. However, these results require further verification.

Radiomics features have been proven to be important markers for tumor heterogeneity by some researchers.^[45]^ The complexity of hierarchical changes within the tumor can be reflected by texture features, whereas first-order features primarily depend on the statistics of intensity information.^[45]^ In the combined radiomics model, there were seven texture features and two first-order features among the nine features in this study. On the other hand, Laplacian of Gaussian filtered images extracted two features, wavelet four features and three features of the original images. The results indicated that the preprocessed image features were more stable than those of the original images.

This study has several limitations. First, population bias may have been introduced by the single-center retrospective design. Second, some patients were not histologically confirmed according to our inclusion criteria. Third, there was no external validation of the radiomic models developed in our study.

In conclusion, the combined radiomics model built by integrating the significant unenhanced and venous radiomics features demonstrates the best performance for differentiating early stage adrenal metastases from LPAs. The combined radiomics model yields an incremental discrimination ability over all the other radiomics models and especially the clinical-radiological model. Thus, it will be a useful and noninvasive tool to identify the early stage adrenal metastases and assist clinicians in pretreatment decision-making.

## Acknowledgments

We thank all the patients enrolled in this study.

## Author contributions

**Methodology:** Wengui Xu.

**Supervision:** Wengui Xu.

**Writing – review & editing:** Lixiu Cao.
